# Passive Way of Measuring QOL/Well-Being Levels Using Smartphone Log

**DOI:** 10.3389/fdgth.2022.780566

**Published:** 2022-03-10

**Authors:** Wenhao Yao, Kohei Kaminishi, Naoki Yamamoto, Takashi Hamatani, Yuki Yamada, Takahiro Kawada, Satoshi Hiyama, Tsukasa Okimura, Yuri Terasawa, Takaki Maeda, Masaru Mimura, Jun Ota

**Affiliations:** ^1^Department of Precision Engineering, School of Engineering, The University of Tokyo, Tokyo, Japan; ^2^Research Into Artifacts, Center for Engineering (RACE), School of Engineering, The University of Tokyo, Tokyo, Japan; ^3^X-Tech Development Department, NTT DOCOMO, Inc., Tokyo, Japan; ^4^Department of Neuropsychiatry, Keio University School of Medicine, Tokyo, Japan; ^5^Department of Psychology, Keio University, Tokyo, Japan

**Keywords:** smartphone, mental health, quality of life, well-being, machine learning

## Abstract

Research on mental health states involves paying increasing attention to changes in daily life. Researchers have attempted to understand such daily changes by relying on self-reporting through frequent assessment using devices (smartphones); however, they are mostly focused on a single aspect of mental health. Assessing the mental health of a person from various perspectives may help in the primary prevention of mental illness and the comprehensive measurement of mental health. In this study, we used users' smartphone logs to build a model to estimate whether the scores on three types of questionnaires related to quality of life and well-being would increase compared to the previous week (fluctuation model) and whether they would be higher compared to the average for that user (interval model). Sixteen participants completed three questionnaires once per week, and their smartphone logs were recorded over the same period. Based on the results, estimation models were built, and the F-score ranged from 0.739 to 0.818. We also analyzed the features that the estimation model emphasized. Information related to “physical activity,” such as acceleration and tilt of the smartphone, and “environment,” such as atmospheric pressure and illumination, were given more weight in the estimation than information related to “cyber activity,” such as usage of smartphone applications. In particular, in the Positive and Negative Affect Schedule (PANAS), 9 out of 10 top features in the fluctuation model and 7 out of 10 top features in the interval model were related to activities in the physical world, suggesting that short-term mood may be particularly heavily influenced by subjective activities in the human physical world.

## 1. Introduction

Over the past few decades there has been an increasing focus on the importance of mental health. Already back in 1992 the World Federation for 23 Mental Health set October 10 the as annual “World Mental Health Day” from 1992 to disseminate accurate information and raise awareness regarding mental health problems (1). Mental health can be compromised by a variety of factors, including intense stress, social isolation, and loneliness. This can lead to sleep disorders, anxiety disorders, impaired attention, depression, adjustment disorders, addiction, and other disorders. These symptoms are assessed using indexes such as the Pittsburgh Sleep Quality Index (2); State-Trait Anxiety Inventory (STAI), used to measure excessive anxiety (3); and the ratio of Low Frequency to High Frequency (LF/HF) index by heart rate R-R interval, used to evaluate stress (4). These indexes, however, measure the state of some aspects of mental health or determine a part of the cause. Using indexes related to quality of life (QOL) and well-being to determine whether a person is healthy in terms of various aspects could help in the primary prevention of mental illness and the comprehensive measurement of mental health.

QOL was defined as “individuals' perception of their position in life in the context of the culture and value systems in which they live and in relation to their goals, expectations, standards, and concerns (5).” Well-being is considered “what is intrinsically valuable relative to someone. So the well-being of a person is what is ultimately good for this person, what is in the self-interest of this person (6).” Subjective well-being and subjective QOL are often used interchangeably in psychological research, policy, and practice (7). For simplicity, in this paper, “QOL” is used interchangeably with “QOL/well-being.”

Studies measuring QOL ask respondents about a variety of items and base their assessment on respondents' answers. Examples include the Positive and Negative Affect Schedule (PANAS) or the Positive and Negative Affect Schedule—Expanded Form (PANAS-X), which comprise two dominant and relatively independent dimensions categorized as positive and negative affect (8, 9); the Flourishing Scale (FS), which assesses psychological flourishing and feelings (10); the Subjective Well-Being Inventory (SUBI), which assesses mental health and fatigue (11); the World Health Organization Quality of Life Assessment (WHOQOL), which assesses individuals' perception of their position in life (5); the MOS 36-item Short-Form Health Survey (SF-36) and its shortened version SF-12, which comprehensively examine eight health concepts (12, 13); and the Satisfaction With Life Scale (SWLS), which assesses life satisfaction (14). However, the burden of administering questionnaires can be difficult to manage.

Smartphones have become widespread because of advances in information and communication technology and the popularity of mobile applications, including social networking services (SNS), e-commerce, and games. Smartphones are an integral part of our daily life. If QOL can be analyzed using the daily activity data of smartphone users, the burden on users can be reduced. However, no studies so far have made it possible to evaluate QOL using only smartphones. Considering that many people use and carry smartphones at all times, if QOL can be evaluated through smartphones, it may prove to be a convenient means to obtain clues for the promotion of self-care, primary prevention of mental illness, and daily monitoring of mental health. Therefore, this study aims to evaluate users' mental state by estimating QOL indexes through smartphone data, generated on a daily basis.

## 2. Literature Review

Smartphones are equipped with sensors, such as accelerometers, pressure sensors, and illuminance sensors, and can record global positioning system (GPS) data and application usage history. Therefore, smartphones can be an important means for obtaining information about individuals' daily activities and their environments.

Conventional studies measure several types of daily indicators based on smartphone and wearable device logs, including stress. Sano et al. (15) classified participants' perceived stress scale as high or low based on smartphone logs, especially focusing on communications, wrist sensors, and surveys. High stress levels were associated with activity levels, short message service usage, and screen on/off. Gjoreski et al. (16) estimated the stress levels of students by assessing their smartphone logs. They showed that the student-specific estimation model performed well. Ferdous et al. (17) estimated the stress levels of participants based on their patterns of using smartphone applications in the workplace. Yamamoto et al. (18) showed that stress expressed in terms of physiological indicators could also be estimated with 71% accuracy using only smartphone logs. Anxiety has also been a focus of several studies. Fukazawa et al. (19) estimated the change in users' anxiety based on STAI with 0.742 F-score using smartphone logs of experimental participants. Boukhechba et al. (20) estimated students' levels of social anxiety based on GPS location, text message, and call data of smartphones and showed the importance of using both behavioral and communication patterns. Saeb et al. (21) evaluated the correlation between GPS features of smartphones and depression and found that information gathered on weekends was more useful than that on weekdays, and Mehrotra (22) succeeded in automatically extracting useful features from raw GPS location data to predict depressive states. Lu et al. (23) showed that location information of smartphones and activity, sleep, and heart rate information of wearable devices can be used to estimate not only self-reported scores of depression severity but also clinical assessments. Exler et al. (24) combined logs of smartphones and smartwatches as wearable heart rate monitors to estimate variability in valence—one of the components of mood—with accuracy of up to 91%. Servia-Rodríguez et al. (25) explored relations between behaviors inferred from smartphone logs and users' demographics and psychological factors and the predictability of users' moods by analyzing smartphone logs and reported mood data collected using a smartphone application published in the Google Play Store. As a result, they were able to predict the user's mood with 68% accuracy, especially during weekends.

In the abovementioned studies, mental states such as stress and emotions have been estimated from smartphone logs, but indicators such as QOL have not been discussed. Amenomori et al. proposed a simple estimation method for QOL by using a smart device (26). They collected GPS information using a smartphone and biometric information—such as acceleration, heart rate interval, and skin body temperature—using a wearable device (i.e., E4 wristband). Subsequently, the WHOQOL (5) questionnaire was used as the ground truth. Sano et al. classified high and low SF-12 (13) and other indicators based on information from a smartphone application that recorded phone usage and geolocation patterns, wrist-based sensors that recorded physical activity and autonomic physiology, and electronic diaries (27, 28). In both studies, the wearable sensor logs performed better than the smartphone logs in estimation. Lathia et al. examined the relation between data on physical activity obtained from self-reports and smartphone accelerometers and happiness [calculated based on questionnaires including the PANAS-X (9) and SWLS (14)] in a large number of users who installed a smartphone application published in the Google Play Store (29). They found that the smartphone accelerometer data could predict both positive and negative affect. The study partially predicted scores on items related to QOL, but not all of the rich information available through smartphones was used.

Thus, most conventional studies on psychological states using smartphone data investigate a single aspect of mental health. In addition, few studies on QOL have achieved excellent estimation by using only smartphones, and they have not identified the types of indicator variation that can be estimated well. Estimating conditions based solely on information from smartphones is highly convenient and could lead to more people gaining the opportunity for self-care. Therefore, in this study, we aimed to build an estimation model for multiple QOL indicators by using only smartphones.

## 3. Problem Setting

**Figure 1:**
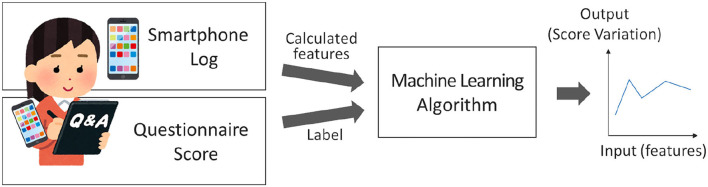
Framework of this study.

The outline of the model proposed in this study is shown in Figure 1. First, the experiment was conducted, and the participants responded to the questionnaires. During the experiment, a smartphone log collection application installed in the participants' smartphones continually collected smartphone logs. Scores were calculated from the answers to the questionnaire, and labeling was conducted. From the collected smartphone logs, features were constructed on a daily and weekly basis. Based on the set of labels and features, estimation models were constructed using the machine learning algorithm.

## 4. Methods

### 4.1. Experiment

To evaluate the proposed model, an experiment was conducted, which was approved by the ethics committee at the School of Engineering, the University of Tokyo (KE20-3). The participants in the experiment were 16 university students aged 20–29 years (13 male and 3 female). The experimental period was 14 weeks: from September 29, 2020, to January 5, 2021.

To begin the experiment, the participants installed a smartphone log collection application. During the experiment period, smartphone logs were continuously recorded. Participants answered three QOL questionnaires every Tuesday. These questionnaires were distributed online, and each participant answered them.

### 4.2. Questionnaire

In this study, participants were asked to answer three questionnaires, namely, the PANAS (8), the FS (10), and the SUBI (11), to assess QOL from different perspectives. The PANAS measures short-term mood more than the other two. The FS complements other measures of subjective well-being and measures social–psychological prosperity. Unlike the FS, the SUBI also considers physical components and assesses positive and negative aspects of the states separately. In our preparatory experiment, we confirmed that there was no significant correlation between the scores of these indexes [The correlation coefficients and *p*-values were 0.315 (*p* = 0.27) between PANAS and FS, 0.224 (*p* = 0.44) between PANAS and SUBI, and 0.281 (*p* = 0.33) between FS and SUBI], and it is valuable to be able to estimate their variations independently.

The PANAS is a self-reported questionnaire comprising two mood scales that measure positive (e.g., feeling enthusiastic, active, and alert) and negative (e.g., feeling distress and dissatisfaction) affect. Each item is rated on a six-point scale from 1 (not applicable at all) to 6 (very well-applied). Kawahito et al. (30) conducted a questionnaire survey on 442 university students to confirm the reliability and validity of the Japanese version of the PANAS. The reported Cronbach's alpha coefficients for the positive and negative affect items were 0.86 and 0.89, respectively.

The FS is an eight-item measure of social–psychological prosperity based on the theory of psychological and social well-being. It is designed to measure social–psychological prosperity and complement existing subjective well-being. Sumi et al. (31) evaluated the reliability and validity of the Japanese version of the FS by using a sample of 520 Japanese university students. The reported Cronbach's alpha coefficient was 0.95, and the scale had acceptable reliability and validity, in line with the original version.

The SUBI attempts to capture well-being from psychological, physical, and social aspects according to two aspects: positive and negative. The choices are described in sentences for each item, such as “I agree very much,” “I agree to some extent,” and “I do not agree so much.” Tonan et al. (32) conducted a Japanese version of the SUBI with 168 male and female college students, 61 adults, and 38 first-visit patients in a psychiatric setting and verified their validity by using comparative analysis. For the seven main factors extracted, Cronbach's alpha coefficients ranged from 0.60 to 0.88.

### 4.3. Feature Extractions From Smartphone Log

Smartphone logs were obtained comprehensively from embedded sensors (excluding, e.g., conversational voice and specific typed text). The following is a description of three types of logs: those reflecting activities in the physical world, those reflecting activities in the cyber world, and those reflecting individuals' environment (Table 1).

**Table 1:** Three type of features extracted from smartphone log.

**Type**	**Features**
	
	Acceleration average
	Maximum acceleration
	Minimum acceleration
	Acceleration maximum/minimum difference
	Acceleration variance
	Tilt average (three directions)
	Tilt variance (three directions)
	Gyro average
	Gyro maximum value
Maximum distance from home	Gyro minimum value
	Gyro maximum/minimum difference
	Gyro variance
	Times of still
	Times of tilting
	Times of foot
	Times of bicycle
	Times of vehicle
	Total distance
	Physical world activity
	
	
	Times of charges
	Screen on/off times
	Earphone connection count
	Amount of battery power used in a day
Cyber world activity	Storage capacity (free)
	Storage capacity (total capacity)
	SNS application times
	Message application times
	Game application times
	Shopping application times
	
	
	Average illuminance
	Maximum illuminance
	Minimum illuminance
	Illuminance maximum/minimum difference
	Illuminance variance
Environment	Average atmospheric pressure
	Maximum atmospheric pressure
	Minimum atmospheric pressure
	Atmospheric pressure maximum/minimum difference
	Atmospheric pressure variance
	Holidays

*The background color of the tables indicates the type of each feature: red for physical world activity, blue for cyber world activity, and green for the environment, respectively*.

The logs reflect physical world activities show smartphone users' actions in the physical world, such as how individuals behave and their movements, based on the accelerometer log and the movement trajectory of the person according to GPS information. The logs that reflect physical world activity are mainly acceleration, tilt, gyro, activity recognition, and position.

Logs that reflect cyber world activity reflect the operation and history of individuals in the cyber world—for example, when they use their smartphone or are online, how often smartphone operations are used by observing when users turn on/off the screen of the smartphone, and how often a game application is used based on the application history. Logs that reflect cyber world activities are mainly application history, the screen on/off, amount of battery power used in a day, number of times the phone is charged, storage, and earphone jack on/off.

Logs reflecting the users' environment show characteristics such as changes in atmospheric pressure and room brightness in the users' environment. Logs that reflect the location environment are mainly illuminance, atmospheric pressure, and whether it is a holiday.

Logs are recorded in 1 Hz for 24 h while the smartphone log collection application runs normally. Missing value imputation and normalization were then performed. The average, maximum value, minimum value, maximum–minimum difference, variance, and the number of times the aforementioned log values occurred in 1 day were calculated and used as features. The total number of features per day was 44. By arranging the features for 7 per week, 308 features were obtained.

Weekly features were also constructed. For 44 features per day, the average and variance for 7 days were calculated for the features other than the dummy variable “holiday,” and a total of 86 features were obtained. Adding these together, a total of 394 features could be created per week.

### 4.4. Estimation Model

In this study, we propose two models—a fluctuation model and an interval model—for QOL. The score is calculated from the participants' answers to the questionnaire. The structure of the model differs according to how this questionnaire score is estimated. The fluctuation model estimates whether the current score will be higher or lower than the prior score. The interval model estimates whether the score is located in the interval above or below the mean.

#### 4.4.1. Setting Ground Truth

When attempting to understand the changes in mental health as a QOL index, whether the next score increases or decreases is important. The fluctuation model estimates the fluctuation status of each score compared with the prior score. In the experiment we conducted, the questionnaires were answered once per week every Tuesday. Therefore, the difference between the score on Tuesday of the relevant week and that on Tuesday of the prior week is considered.

**Figure 2:**
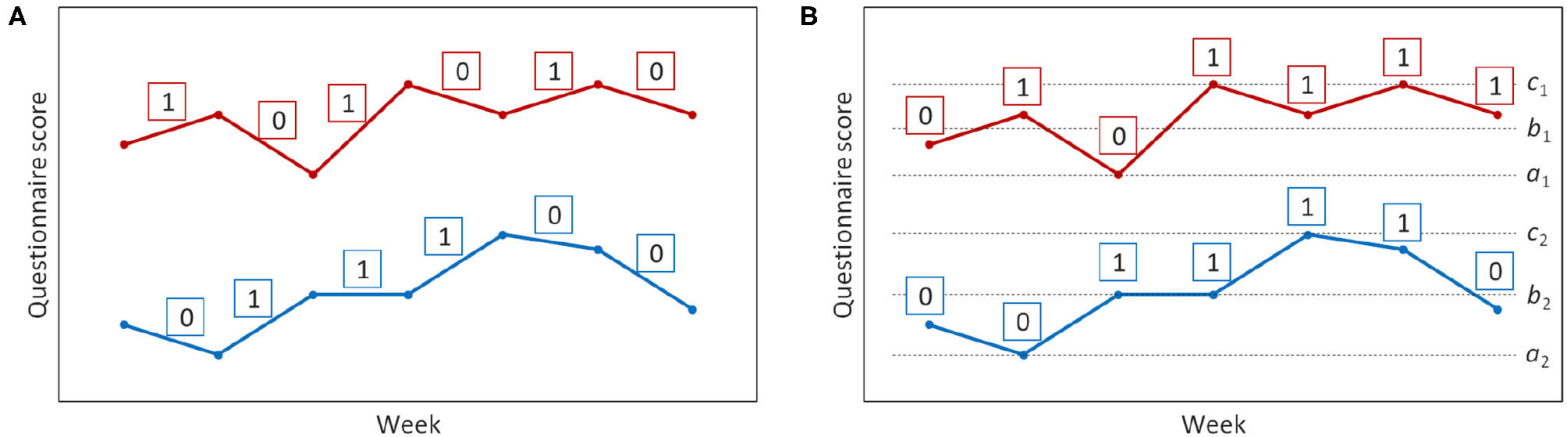
Label of each estimation model. **(A)** Label of fluctuation model. **(B)** Label of interval model. The questionnaire scores of two different users are shown in different colors. *a*_1_ and *c*_1_ are the minimum and maximum scores of one user, *a*_2_ and *c*_2_ are the minimum and maximum scores of another user over the whole period. *b*_1_ and *b*_2_ are the average of *a*_1_ and *c*_1_, *a*_2_ and *c*_2_, respectively.

The user's QOL indexes were classified into two scenarios: not changing or increasing and decreasing. Specifically, if the score for a questionnaire is higher than or same as the prior score, label 1 was used, and if the score is lower, label 0 was used (Figure 2A). As a result, the fluctuation estimation problem resulted in a two-classification problem. At week *i*, ground truth *y*_*trace*_*i*_ was labeled as follows:


(1) $$\begin{array}{l} y_{trace\_i}=\left\{ \begin{array}{l} 1\;;\;score_{i}\geq score_{i-1}\;\\ 0\;;\;score_{i}<score_{i-1} \end{array}\right. \end{array}$$

Each individual has a different range of possible mental health scores. Knowing whether a user's current state is not worse than the average state, which varies from individual to individual, can be important for early detection of a worsening state. Such an estimation model, when used in conjunction with the fluctuation model, may be useful in understanding conditions such as the user's score being lower than usual and in the direction of further decline. This concept is similar to that posited by Yamamoto et al. (18) who evaluated how good the stress-related LF/HF of heart rate was from an individual's average.

Here, unlike the fluctuation model, which estimates the result of comparing the current score with the previous score, the interval model estimates whether the current score is higher than a certain threshold. The case in which the score interval of the questionnaire over the whole period is [*a, c*] for a certain user is considered. The threshold value is set as the average value *b* of *a* and *c*. The interval [*a, b*] is named L-QOL (Low QOL Level), and [*b, c*] is named H-QOL (High QOL Level).

Label 1 for the score in the H-QOL section and label 0 for the score in the L-QOL section (Figure 2B). As a result, the interval estimation problem can be reduced to a two-classification problem in the same manner as the fluctuation estimation problem. The label *y*_*range*_ is as follows:


(2) $$\begin{array}{l} y_{range\_i}=\left\{ \begin{array}{l} 1\;;\;score\geq average(a,c)\;\\ 0\;;\;score<average(a,c) \end{array}\right. \end{array}$$

#### 4.4.2. Machine Learning Algorithm for Estimation

The fluctuation model and interval model of this study were constructed using random forests (33), an ensemble learning model that combines multiple decision trees. Random forest has two advantages over other learning algorithms. First, it has a multiple tree structure, which is advantageous for managing problems with many variables. The maximum number of features in this study is 394; therefore, it can benefit from this method. Second, we can also calculate which variables are important for the classification result. This study attempts to analyze which smartphone log features are useful for estimating the QOL index. Third, several related studies have shown the high estimation performance of random forests (16, 19, 26, 34). For these reasons, random forests are used in this study. The hyperparameters of the random forest were set as follows: n_estimaters were 100 for the PANAS, 500 for the FS, and 100 for the SUBI in the fluctuation model and 100 for the PANAS, 100 for the FS, and 100 for the SUBI in the interval model. The criterion was set as gini, and max_depth was set as None. The hyperparameters were manually adjusted for better performance on the training data.

Leave-one-participant-out cross-validation and F-score were used to train and verify the data. In other words, the data of one participant was excluded from the training when estimating participants' scores. F-score is the harmonic mean of the precision (the proportion of what is truly positive that is estimated to be positive) and the recall (the proportion of the data estimated to be positive that is truly positive).

## 5. Results

In this experiment, 16 university students were tested for 14 weeks, and 224 pieces of data were obtained for each questionnaire. Using random forest, a fluctuation model was created to estimate whether the questionnaire score had increased compared to the previous week and an interval model to estimate whether the score was higher than the average of the individuals' scores from the smartphone logs.

### 5.1. Performance of Estimation

**Figure 3:**
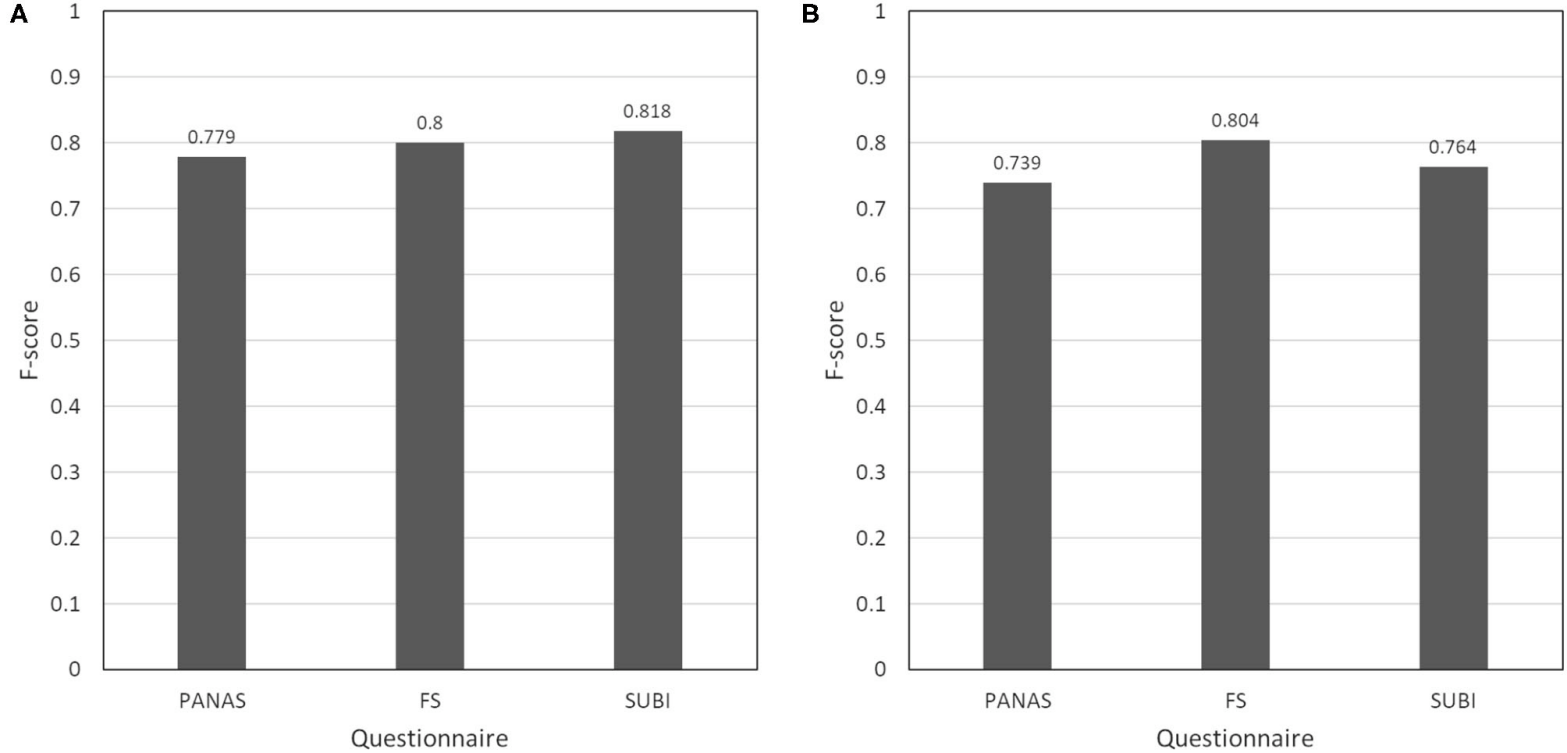
F-score at each condition. **(A)** F-score of fluctuation model. **(B)** F-score of interval model.

To evaluate the performance of each estimation model, F-score was calculated based on the estimation results (Figure 3). The F-score of the fluctuation model was 0.779 for the PANAS, 0.800 for the FS, and 0.818 for the SUBI (Figure 3A). The F-score of the interval model was 0.739 for the PANAS, 0.804 for the FS, and 0.764 for the SUBI (Figure 3B).

### 5.2. Top Features of Each Questionnaire

To understand which smartphone log features the estimation models were focusing on to make estimations about the questionnaire scores, we selected the top 10 features by using Gini coefficient reduction. The selected top features of the estimation models in the PANAS, FS, and SUBI are presented in Tables 2–**4**, respectively. The background color of the tables indicates the type of each feature: red for physical world activity, blue for cyber world activity, and green for the environment, respectively. The plus and minus signs next to the feature name indicate the relationship between the feature and the label. They were described based on the values obtained by dividing the data set into two groups, labeled 0 and 1 and calculating the average value of the feature in each group. Label 1 indicates that the questionnaire score has increased from the previous week in the fluctuation model and that the questionnaire score is above average in the interval model. For example, in Table 2, the minus sign next to average illuminance indicates that when the label is 1, the value of average illuminance is smaller than when the label is 0. This means that if the questionnaire score went up from the previous week, the value of average illuminance, which was focused on by the estimation model, was smaller than if the survey score went down from the previous week. Note that what is shown here is only the difference in the average of the feature value between the case with label 1 and the case with label 0. It does not compensate for the existence of a simple relationship, such as the label being 1 when the value of the feature is large.

**Table 2:** Top 10 features of the PANAS.

	**Fluctuation model**		**Interval model**	
1	Average illuminance	−	Tilt average (x direction)	+
2	Minimum acceleration	−	Maximum illuminance	+
3	Tilt average (y direction)	+	Times of Foot	+
4	Tilt variance (z direction)	+	Acceleration max/min difference	+
5	Gyro max/min difference	+	Gyro minimum value	+
6	Gyro minimum value	−	Tilt average (z direction)	-
7	Tilt variance (x direction)	+	Illuminance variance	+
8	Times of still	+	Gyro minimum value	+
9	Gyro maximum value	−	Tilt variance (y direction)	-
10	Tilt variance (x direction)	−	Minimum illuminance	−

*The background color of the tables indicates the type of each feature: red for physical world activity, blue for cyber world activity, and green for the environment, respectively*.

With regard to the top features of the PANAS, most are related to physical world features; this indicates that the models estimated for the PANAS scores were more focused on features related to the physical world, such as accelerations and tilts of the smartphones. With regard to the top features of the FS, almost half are related to physical world activity, and the other half are related to the environment, such as atmospheric pressure and illuminance. As for the top features of the SUBI, most features in the fluctuation model are related to the environment in the interval model or related to physical world activity.

## 6. Discussion

This study estimates the score variability of the QOL questionnaire with an F-score ranging from 0.739 to 0.818. In the study by Fukazawa et al. (19) that predicted the anxiety state, F-score ranged from 0.702 to 0.742. Although a simple comparison cannot be made because of the differences in tasks, the performance of the estimation models created in this study is comparable to their study. The performance and top features differ for different types of estimation models and questionnaires.

### 6.1. Differences in Top Features

#### 6.1.1. Top Features for Each Questionnaire

In the PANAS, 9 out of 10 features in the fluctuation model and 7 out of 10 features in the interval model are related to physical world activities (Table 2). The PANAS is a questionnaire that mainly asks about short-term moods, such as feelings (e.g., joy, depression, and sadness). Therefore, subjective activities in the physical world of human beings, such as movement and walking, are considered to mainly affect short-term moods, such as those assessed by the PANAS. Budner et al. (34) estimated user emotions by using a smartwatch and found that the characteristics highly correlated with human pleasure and activity were values related to human activity and GPS. In this result, the gyro and acceleration appear in the features that reflect higher physical world activities. The gyro and acceleration are features that reflect the movement of the smartphone and the smartphone owner to some extent. Therefore, the results of this study are similar to those of Budner et al. The relationship between feature values and label changes is not consistent across models. When the extent of motion is high, the scores tend to be lower than the previous week, but better than the average. This suggests that the short-term relationship between feature values and scores on motion does not necessarily match the long-term relationship.

In the FS estimation model, environmental features such as atmospheric pressure and illumination were calculated to be the top features (Table 3). Past studies have proposed that the environment, especially the atmospheric pressure around individuals, influences their thinking regarding life, which also means a change in values. According to another study, sympathetic nerves or other chronic pain may be stimulated by low pressure (35). The common relationship between the value of the feature and the increase or decrease of the label in the fluctuation and interval models is that when the average atmospheric pressure is high, the score goes up.

**Table 3:** Top 10 features of the FS.

	**Fluctuation model**		**Interval model**	
1	Gyro max/min difference	−	Acceleration average	+
2	Gyro maximum value	+	Average atmospheric pressure	−
3	Gyro variance	+	Tilt average (z direction)	−
4	Maximum atmospheric pressure	−	Atmospheric pressure variance	−
5	Atmospheric pressure variance	−	Tilt variance (y direction)	+
6	Tilt variance (y direction)	−	Atmospheric pressure max/min difference	+
7	Average atmospheric pressure	+	Holidays	−
8	Amount of battery power used in a day	+	Tilt variance (z direction)	−
9	Minimum illuminance	+	Times of Still	+
10	Tilt variance (z direction)	−	Illuminance variance	+

*The background color of the tables indicates the type of each feature: red for physical world activity, blue for cyber world activity, and green for the environment, respectively*.

The SUBI mainly has questions regarding life satisfaction, accomplishment, relative support, social support, and family relationships. It has the highest number of questions on external (interaction with others) factors among the questionnaires used in this study. Therefore, before the analysis, we predicted that features related to cyber world activity—such as the use of messaging and SNS applications, which reflect interpersonal relationships—would be important for the estimation. However, in the present results, only two features related to cyber world activities related to interpersonal relationships appeared in each estimation model (SNS application times and game application times, Table 4). In other words, physical world activity and environment were more closely related to QOL than cyber world activity. Notably, the scores of all apps tend to decrease when the usage time is long, and it seems that the use of apps does not necessarily lead to an improvement in QOL. The situation with regard to interpersonal relationships—such as the sense of unity with family, relationships with friends, and human relationships at work—constitutes a high proportion of communication in the physical world and tends not to be directly reflected in message and SNS applications. The fluctuation model focuses on features related to environments, and the interval model focuses on features related to physical world activities. Whether the score was higher than that of the prior week was related to environments, and whether the score was higher than normal was related to physical world activity.

**Table 4:** Top 10 features of the SUBI.

	**Fluctuation model**		**Interval model**	
1	Total distance	+	Average atmospheric pressure	−
2	Average illuminance	−	Game application times	−
3	Average atmospheric pressure	−	Acceleration max/min difference	−
4	Minimum atmospheric pressure	−	Tilt variance (x direction)	−
5	Illuminance variance	−	Times of tilting	−
6	Average illuminance	+	Tilt variance (x direction)	−
7	SNS application times	−	Minimum acceleration	+
8	Gyro variance	−	Gyro minimum value	+
9	Maximum illuminance	+	Maximum acceleration	+
10	Average atmospheric pressure	−	Acceleration max/min difference	+

*The background color of the tables indicates the type of each feature: red for physical world activity, blue for cyber world activity, and green for the environment, respectively*.

In the PANAS, which assesses relatively short-term changes in QOL, physical world activity was emphasized; in the FS, which assesses values that are less volatile than those of the PANAS, environments were given more weight. Considering these factors, it is possible that the simple notion that short-term factors are reflected in physical world activities and long-term factors are reflected in environments may not hold true for changes in QOL.

#### 6.1.2. Importance of Accelerometer-Related Features

The top features include features related to movement, such as acceleration, especially for the interval model. In a study by Kiriu et al. (36) on smartphone conversations, when estimating conversation time, features related to acceleration such as standard deviation, maximum value, and maximum/minimum differences of acceleration appeared among the upper features. The focus of their study was on conversation, but considering that conversation reflects interpersonal relationships to some extent, this study's result that the upper features of the estimation results have a large acceleration is consistent with Kiriu et al.'s results. Therefore, features that reflect physical world activities such as acceleration have a high correlation with external (interaction with others) factors. Additionally, in Amenomori et al.'s (26) study, acceleration information was regarded as a higher feature quantity. In their study, acceleration information was acquired by the accelerometer of the smartphone or another wearable device, and there was a difference that the information on the user's movement could be obtained for a longer period of time. However, it is consistent with the conclusion that acceleration reflects the user's movements to some extent and is effective in estimating QOL.

### 6.2. Limitations

Sixteen university students participated in the experiment conducted in this study. The number of participants is not as large as in other studies, and it is unclear whether the same analysis can be conducted for users with varied attributes (e.g., occupation). To further validate the effectiveness of the method, it is necessary to conduct experiments with a larger number of people with more attributes.

We analyzed the features that contribute to the estimation. However, the study only examined the relationship between the features and the labels. We do not know how behavioral changes actually affect QOL. Further research will provide clues regarding the kinds of behavior that encourage users to maintain a good QOL level.

## 7. Conclusions

This study's objective was to evaluate the mental state of users by estimating QOL indexes from information on smartphones that they use on a daily basis. The proposed method had a maximum F-score of 0.818 (fluctuation model, SUBI) and 0.804 (interval model, FS). The results demonstrate that QOL levels can be estimated using smartphone logs. The model for estimating the variability of the three types of questionnaire scores was weighted by different features. Estimation models such as the one proposed in this study may provide a means of self-care and primary prevention of mental disorders for many people who use smartphones on a daily basis.

## Data Availability Statement

The datasets presented in this article are not readily available because, due to the nature of this research, participants of this study did not agree for their data to be shared publicly, so supporting data is not available. Requests to access the datasets should be directed to kaminishi@race.t.u-tokyo.ac.jp.

## Ethics Statement

The studies involving human participants were reviewed and approved by the University of Tokyo Ethics Committee. The patients/participants provided their written informed consent to participate in this study.

## Author Contributions

WY, KK, NY, TH, YY, TK, SH, TO, YT, TM, MM, and JO: conceptualization and methodology. WY, NY, and TH: software and data curation. WY: validation and formal analysis. WY and KK: investigation, resources, writing—original draft preparation, and visualization. NY, TH, YY, TK, SH, TO, YT, TM, MM, and JO: writing—review and editing and funding acquisition. JO: supervision and project administration. All authors have read and agreed to the published version of the manuscript.

## Funding

This study was partially funded by the company NTT DOCOMO, Inc.

## Conflict of Interest

NY, TH, YY, TK, and SH were employed by NTT DOCOMO, Inc. TM and JO have received research support from NTT DOCOMO, Inc. for this work. The remaining authors declare that the research was conducted in the absence of any commercial or financial relationships that could be construed as a potential conflict of interest.

## Publisher's Note

All claims expressed in this article are solely those of the authors and do not necessarily represent those of their affiliated organizations, or those of the publisher, the editors and the reviewers. Any product that may be evaluated in this article, or claim that may be made by its manufacturer, is not guaranteed or endorsed by the publisher.
